# Prevalence, Patterns and Correlates of Cigarette Smoking in Male Adolescents in Northern Jordan, and the Influence of Waterpipe Use and Asthma Diagnosis: A Descriptive Cross-Sectional Study

**DOI:** 10.3390/ijerph110909008

**Published:** 2014-09-01

**Authors:** Nihaya Al-Sheyab, Mahmoud A. Alomari, Smita Shah, Patrick Gallagher, Robyn Gallagher

**Affiliations:** 1 Faculty of Nursing, Jordan University of Science and Technology, Irbid 22110, P.O. Box 3030, Jordan; 2 Faculty of Health, University of Technology, Sydney, Sydney 2007, Australia; E-Mails: Patrick.gallagher@uts.edu.au (P.G.); robyn.gallagher@uts.edu.au (R.G.); 3 Charles Perkins Centre and Faculty of Nursing and Midwifery, the University of Sydney, Sydney 2007, Australia; 4 Faculty of Medical Sciences, Jordan University of Science and Technology, Irbid 22110, Jordan; E-Mail: alomari@just.edu.jo; 5 Faculty of Medicine, Western Clinical School, The University of Sydney, Sydney 2007, Australia; E-Mail: smita_shah@health.nsw.gov.au

**Keywords:** adolescent, asthma, cigarette smoking, prevalence, water pipe, Jordan

## Abstract

Our study investigates the prevalence, patterns and predictors of tobacco smoking among early adolescent males in Northern Jordan and whether asthma diagnosis affects smoking patterns. A descriptive cross sectional design was used. Males in grades 7 and 8 from four randomly selected high schools in the city of Irbid were enrolled. Data on waterpipe (WP) use and cigarette smoking patterns were obtained (*n* = 815) using a survey in Arabic language. The overall prevalence of ever having smoked a cigarette was 35.6%, with 86.2% of this group smoking currently. Almost half of the sample reported WP use. The most common age in which adolescents started to experiment with cigarettes was 11–12 years old (49.1%), although 10 years was also common (25.3%). Significant predictors of male cigarette smoking were WP use (OR = 4.15, 95% CI = 2.99–5.76), asthma diagnosis (OR = 2.35, 95% CI = 1.46–3.78), grade 8 (OR = 1.52, 95% CI = 1.10–2.11), and having a sibling who smokes (OR = 2.23, 95% CI = 1.53–3.24). However, this cross-sectional study cannot establish causality, thus longitudinal studies are needed. Public health programs and school-based anti-tobacco smoking interventions that target children in early years at high schools are warranted to prevent the uptake of tobacco use among this vulnerable age group. High school students with asthma should be specifically targeted.

## 1. Introduction

Tobacco use increases morbidity and mortality [1] and is an established health hazard as it contributes to multiple diseases, including cardiovascular, pulmonary, musculoskeletal, immune diseases, and cancer and asthma [2,3,4,5] Such tobacco-related morbidity occurs disproportionately in Arabic-speaking countries [1], where smoking has reached epidemic rates [6], particularly in men [7]. Tobacco consumption rates in Jordan and the neighboring countries are amongst the highest rates in the world, and of concern given forecasts of further increases in the next few years [6]. However, it is the growth in smoking in adolescents in Jordan which is most concerning. Aside from some fluctuations, adolescent smoking rates have increased from 18% in 1999 to 45% in 2010, with this increase in starting smoking behavior most evident in males [8,9].

While cigarette smoking is a major health issue, waterpipe (WP) use is a growing concern as the use of these two tobacco products is strongly related. WP use is a problem because of the continuity with smoking and it has become an integral part of smoking behavior patterns [9]. Recent cross sectional and longitudinal studies among Arab youth [10] and Jordanian adolescents [9,11] found that WP smoking predicts later cigarette smoking, confirming the gateway hypothesis, although the reverse could also be true [9,11]. Like cigarette smoking, WP use has increased considerably among adolescents in Jordan [9,11], with one fifth of 13–15 years old adolescents being regular WP smokers (smoke at least once/week) [12]. WP smoking seems to have similar negative health affects to cigarette smoking [2], although this has not been well explored.

Regardless of the type of tobacco product, starting smoking during adolescence is an especially important health challenge because of the potential for longer consumption of tobacco across a lifetime and the related negative health impacts [13]. The age at which people start to smoke is crucial [14], as once smoking is commenced the addictive effect of nicotine is likely to promote continued smoking [15,16]. Therefore the age of initiation of smoking often reflects the duration of lifetime tobacco exposure and the intensity of adverse health effects [17,18]. Smoking usually begins in adolescence [19] and the start and maintenance of smoking at this age is strongly influenced by peer pressure [20]. Adolescents seek approval from their peers and tend to adopt risky behaviors to feel that they “fit in” and belong to a group [21,22]. Adolescents who have at least one close friend who is a smoker are much more likely to smoke themselves [23,24]. However, parents, social norms [25] and the school environment can also influence cigarette smoking [22]. Initiation of smoking is more likely in a smoking-tolerant school, in which teachers smoke, [23,26] and adolescents with high exposure to household smoking were more likely to begin smoking [23,27].

All mentioned social factors can override concerns with worsening health problems such as asthma in adolescents [28,29]. The relationship between cigarette smoking and asthma among young people is most likely complex, multi-factorial, and remains to be determined. There is evidence that cigarette smoking may cause or make asthma worse [30,31,32,33,34,35,36], despite this, most cross-sectional studies have found that young people with asthma are also more likely to smoke [24,28,37,38,39]. The situation is not clear cut, as on one hand several longitudinal studies report no differences in smoking onset in adolescents regardless of the presence of asthma [35,40,41,42], and on the other hand other studies report that adolescents with asthma were more likely to become regular smokers [43], especially those with poorer treatment adherence [44] and severe symptoms [43]. It is highly likely then that other factors beyond asthma and smoking which were not measured in these studies may influence this relationship. Furthermore, the relationships have been primarily explored in cross sectional studies and therefore causation cannot be implied. Regardless, any study that examines smoking in young people should consider the presence of asthma. In Jordan, cigarette smoking rates are as high as 29% in adolescents who have asthma [45], however, to the researchers’ knowledge, there is no data is available about the prevalence of WP use among adolescents with asthma in Jordan. Combined with the high prevalence of asthma (17%) among adolescents in Jordan, the added impact of smoking could be extensive and a focus on students who have asthma is crucial [46].

Systematic approaches to reducing tobacco use in Arabic countries such as Jordan need to be implemented to reduce smoking in adolescents and the associated health burden, particularly in relation to asthma. Effective policies and interventions depend on a clear understanding of the patterns of tobacco consumption including frequency, intensity, and age of initiation as well as influential and predictive factors. The current study aims to identify the prevalence, patterns, and predictors of cigarette smoking and the prevalence of waterpipe use in early male adolescents in Northern Jordan.

## 2. Experimental Section

### 2.1. Study Design

A descriptive cross sectional design was used with early adolescent participants, aged 12–13 years old (mean age was 12.5 years) drawn from four high schools for males. In this study, the term “early adolescent” was defined as any student enrolled in grade 7 and 8 in one of the eligible high schools for males in Irbid city.

### 2.2. Setting and Sample

The schools were randomly selected from all 21 public high schools for males in the first educational district of the greater Irbid area. Irbid was selected because previous studies indicated a high prevalence of smoking in adolescents [11,47]. The schools were randomly selected from all-boys schools in Irbid city using an opaque envelope technique and represent a diverse sample and included both inner city and suburban areas, differing socioeconomic levels and smoking policies, with one school supported by USAID, which has strict anti-smoking policy including surveillance cameras. This school was among participating schools. All school administrations provided approval and Human Research Ethics Approval was obtained from all institutions (Jordan University of Science and Technology and Ministry of Education, Irbid District, Jordan). The required minimal sample was determined to be 818 on the basis of a small effect size (0.02), power of 0.8, 10 predictors in the analyses and a probability level of 0.05 [48]. The four schools had a total population of 882 in grades 7 and 8 (school A = 120, B = 307, C = 215, D = 240). Of these students, 815 (92.4%) participated, as 31 were absent on data collection days and 36 did not return a signed parental consent form.

### 2.3. Instruments

Data were collected using an anonymous survey which included a demographic checklist, Youth Risk Behaviour Survey (YRBS) on cigarette smoking and waterpipe use and the International Study of Asthma and Allergies in Childhood (ISAAC) questionnaire on asthma. Demographical data was obtained from students using an instrument developed previously for this purpose and included age, grade, and school [46].

#### 2.3.1. Tobacco Use

The prevalence, pattern, and predictors of student smoking behavior were all assessed using a survey in Arabic language that was adopted from the YRBS. The Centers for Disease Control and Prevention developed the YRBS to monitor six categories of priority health-risk behaviors among youth including tobacco use [49]. Ten questions were taken from the YRBS. Five questions addressed the pattern of students’ active smoking behavior and included two “Yes/No” questions about the prevalence of cigarette and waterpipe smoking, one about the frequency of cigarette use (number of days smoked) in the past month, ranging from none to all days of the month, one about the total number of cigarettes per day (intensity) in the past month ranging from none to more than 20 cigarettes/day, and one about the age of initiation of cigarette smoking (ranging from less than or equal to eight years to 13–14 years old). The definitions of smoking behaviours were classified according to Table 1 [9]. One question asked the number of days in a week that students are exposed to passive cigarette smoking (ranging from zero to seven days) and another four “Yes/No” questions asked about the prevalence of student household smoking (father, mother, sibling). The English version of the YRBS has been reported to be reliable [50,51].

**Table 1:** Definitions of terms [9].

Term	Definition
Ever cigarette smoking	ever smoking a cigarette, even a puff or two
Current cigarette smoking	used cigarette in the past 30 days
Regular cigarette smoking	smoking at least once a week in the past 30 days
Never cigarette smoking	never having experimented with cigarettes

The main outcome in this study is ever cigarette smoking defined as “ever smoking a cigarette, even a puff or two”. The researchers decided to use this definition given the very young age (mean 12.5 years old) of the sample, as these junior high school students may still be experimenting with tobacco products. The focus in this age group is to stop any smoking behaviour before it becomes a regular habit [52]. In this respect, ever cigarette smoking is indicative of smoking initiation, whereas current smoking is an indicator of an established smoking habit. Adolescents who start smoking early, even occasionally, are highly susceptible to become daily smokers later with smoking even one cigarette predicting subsequent use [53], which may be especially the case when WP use is prevalent [9,12].

#### 2.3.2. Asthma

Students were screened for asthma and recent asthma symptoms using the Arabic version of the ISAAC Written Questionnaire. This tool was developed to identify children likely to have asthma at young ages by taking a symptomatic not diagnostic approach [54]. All students who answered yes to the question “have you ever had asthma?”, reported recent symptoms during the last 12 months, and their school files, when reviewed by the researchers, confirmed current asthma diagnosis were considered as having recent asthma diagnosis. The ISAAC written questionnaire was reported to be valid in young people internationally [55] and in a similar population previously [45]. The internal consistency “reliability” of the ISAAC written questionnaire was found to be adequate in this study (Cronbach’s Alpha = 0.82). In this study, adolescents with “asthma symptoms only” were defined as those who had reported recent symptoms in the last 12 months but were never diagnosed by healthcare professionals, thus they are not aware that they have had asthma. Those adolescents were identified when they answered yes to the question “have you had wheezing during the last 12 months?” This definition was also used in similar previous research [46].

### 2.4. Procedure

Data collection was conducted from November 2012 to May 2013 from participating students in the selected schools. Students from grades 7 and 8 were approached to join the study through school principals and volunteer teachers in students’ classes. All students who agreed to participate, and who received their parents’ written consent to participate completed the survey at school, which took 10 min to complete. A teacher and two members of the research team were available for any questions from participants.

### 2.5. Data Analysis

Data were entered into and analyzed using SPSS version 22. Missing data was rare and resulted in removal of the participant. Frequencies and percentages, means and standard deviations were used to describe the sample. Chi-squared analyses were used to compare subgroups of dichotomous variables; those who had ever smoked *versus* those who had never smoked cigarettes on school year (grade), school type (socioeconomic status), asthma diagnosis, family smoking (exposure to smoking), and waterpipe use, as illustrated in Table 3. The potential independent predictors were selected based on their association with the main outcome of this study as confirmed in the literature and Chi-squared analyses (Table 3). These independent predictors of ever having smoked were determined using logistic regression analysis. Potential variables were tested for collinearity, which was examined in this regression model through Variance Inflation Factor (VIF), and there were no values >2, indicating low level of multicollinearity. The variables included in the analyses were school grade, school, family member (mother, father, sibling) smoking, waterpipe smoking and asthma diagnosis. The critical level was set at 0.05.

## 3. Results

### 3.1. Participants

The average age of the sample was12.5 years; 49% were in grade 7 and 51% in grade 8, with 97 out of 815 (11.9%) having recent asthma diagnosis (in the last 12 months).

### 3.2. Prevalence and Patterns of Tobacco Use

The overall prevalence of ever smoking was 35.6% (*n* = 290), of this proportion 86.2% (*n* = 253) were current smokers (Table 2). Most of the participants who had ever smoked had begun smoking by 12 years of age (74.4%), with more than 25% having begun smoking by 10 years of age. The most common frequency of smoking was up to 5 cigarettes/day, however 17.9% were smoking at least two packets of cigarettes per week. Waterpipe use was also very common, with almost half of the sample reporting use (48.6%, *n* = 396) and 25.6% (*n* = 209) using both waterpipe and cigarettes. Exposure to smoking was high with 66.1% (*n* = 504) of the males living with a family member, who was most often their father at 48.0% (*n* = 391).

**Table 2:** Patterns of smoking in those students who have ever smoked (*n* = 290).

Characteristic	N	% *
Age of smoking initiation		
≤8 yrs	31	11.7
9–10 yrs	36	13.6
11–12 yrs	130	49.1
13–14 yrs	68	25.7
Days smoking in last month		
0 days	34	12.6
1–9 days	186	69.2
10 to 19 days	29	10.8
≥20 days	20	7.4
Number of cigarettes smoked/day in last month		
Nil	37	13.8
1–5 cig./day	181	67.6
6 to 10 cig./day	26	9.7
11 to 20 cig./day	22	8.2

* Percentages may not add to 100 due to missing values.

We compared those students who had never smoked with those who had tried smoking (Table 3). Statistically significant differences occurred for grade, school, asthma diagnosis, sibling smoking and waterpipe use. More students in grade 8 (59%) reported ever smoking cigarettes compared to those in grade 7 (41%) (*p* < 0.001). School B had a higher percentage of ever smokers compared to the other schools (40% *versus* 26%, 19% and 16%, *p* < 0.01). Students with diagnosed asthma were twice as likely to report ever smoking than never smoking cigarettes (19% *versus* 8%, *p* < 0.001), whereas there were no statistically significant differences of ever and never smoked among students who reported asthma symptoms only. Interestingly, students with an asthma diagnosis reported higher rates of “ever” cigarette smoking compared to those with symptoms only (19% and 11% respectively). Students who had siblings who smoked were more likely to ever smoke (34% *versus* 15%, *p* < 0.001). Importantly, students who used a waterpipe were twice as likely to ever smoke than non-users (72% *versus* 36%, *p* < 0.001).

**Table 3:** Baseline characteristics compared for students who had ever smoked *versus* those who had never smoked cigarettes.

Characteristic	Had Smoked	Had Never Smoked	Chi-sq (*p*)
	(*n* = 290)	(*n* = 525)	
	N (%)	N (%)	
School year Grade 7 Grade 8	118 (41%) 172 (59%)	281 (54%) 244 (46%)	11.81 (<0.001)
School A B (Low SES **) C (USAID) D	45 (16%) 115 (40%) 54 (19%) 76 (26%)	68 (13%) 183 (35%) 154 (28%) 120 (23%)	11.33 (0.01)
Asthma Diagnosis No asthma Diagnosed asthma Symptoms only	204 (70%) 54 (19%) 32 (11%)	412 (78%) 43 (8%) 70(13%)	19.50(<0.001)
Exposed to smoking ≥1 day/week *	214 (74%)	345 (66%)	5.29 (0.10)
Water-pipe use	209 (72%)	187 (36%)	97.90 <0.001)

* Numbers represent students who only reported at least one family member who is a smoker, students may have reported more than one family member, ** Socioeconomic status.

### 3.3. Correlates of Students Reporting Ever Having Smoked Cigarettes

To identify the independent predictors of smoking we conducted logistic regression analyses on the total sample (Table 4). The model developed identified four variables that independently predicted (statistically significant) ever having smoked (Chi-squared test = 150.73, *p* < 0.001). The odds of a student answering yes to being a cigarette smoker was more than four times higher for waterpipe users (OR = 4.15, 95% CI = 2.99–5.76), more than twice as high for students with an asthma diagnosis (OR = 2.35, 95% CI = 1.46–3.78) or those who had a sibling who smoked (OR = 2.23, 95% CI = 1.53–3.24) and more than 50% higher in grade 8 students than those in grade 7 (OR = 1.52, 95% CI = 1.10–2.11).

**Table 4:** Predictors of students ever having smoked cigarettes.

Predictors	Odds Ratio	95% CI ^d^	*p*-Value
Uses waterpipe	4.15		<0.001
Diagnosed with asthma	2.35	2.99–5.76	<0.001
Sibling smokes	2.23	1.46–3.78	<0.001
Grade 8 student	1.52	1.53–3.24	0.011
School C * (*vs* C)	0.63	1.1–2.11	0.09
School B ** (*vs* C)	0.95	0.37–1.08	0.85
School D (*vs* C)	0.98	0.57–1.6	0.94
Mother smokes	0.92	0.58–1.67	0.75
Father smokes	0.96	0.54–1.57	0.8
Constant	0.18	0.69–1.33	<0.001

Model Statistics Chi-squared test = 150.73, *p* < 0.001, ^d^ Confidence Interval, * USAID school, ** low SES.

Type of “ever” tobacco use was also assessed among participating students (Table 5). About fifth of the sample (25.6%) reported smoking both cigarettes and waterpipe.

**Table 5:** Type of “ever” tobacco use among participating students.

Type of Tobacco Use	Frequency (N)	Percentage (%)
Neither (no cigarettes, no waterpipe)	338	41.5
Cigarettes only	81	9.9
Waterpipe only	187	22.9
Cigarettes + Waterpipe	209	25.6

## 4. Discussion

We investigated the prevalence, patterns, and predictors of cigarette smoking behavior among early adolescent males (grade 7 and 8) in high schools for males in Northern Jordan. Our study confirms the strikingly high rates of tobacco use in this age group [11], and draws attention to the sustained increase in rates of cigarette smoking and waterpipe use among Jordanian early adolescent males [9]. This study also found that several variables can predict “ever” cigarette smoking among male Jordanian adolescents. The strongest predictor was WP use, followed by asthma diagnosis, sibling smoking, and finally grade 8.

Our study found that the majority of males who had tried smoking were also current smokers and half of them were regular smokers. Furthermore, current smokers were very regular smokers, consuming up to two packets of cigarettes per week. An important proportion also smoked up to three packets of 20 cigarettes a week, and this intensity is higher than that reported for adolescents from non-Arab countries [56]. The high and regular consumption of cigarettes could seriously affect these young people’s health and could lead them into being regular addicted smokers in adulthood [15,16,57,58]. Moreover, starting tobacco smoking during childhood or early adolescence stage could lead to addiction to tobacco smoking in adulthood [15]. This could be true in our population as well in which a higher prevalence of tobacco initiation and consumption is expected to rise among middle adolescence (grade 9). Therefore, interventions aiming at preventing smoking behavior should include children in grade 7 and those younger than 12 years old and their families to help fight the rapid increase in smoking prevalence among youth before they initiate this risky behavior [59].

The study suggests that there are several factors associated with cigarette smoking behavior among early male adolescents in Jordan. The strongest predictor found in our study was WP use, which was highly prevalent and presents a major health issue. In our study, more than a fifth of the participants used waterpipe only, and this is consistent with previous studies worldwide [60,61,62] and locally among this age group [63]. Congruent with a recent longitudinal study in Jordan, [9] our study showed that waterpipe use during early adolescence was a predictor for cigarette smoking, which could be likely due to nicotine addiction [15,57,58]. Our study, although with cross-sectional design, is therefore one of the first to support the “gateway” hypothesis indicating that one tobacco smoking form will lead to another [9]. This means that the increasing prevalence of waterpipe use is a major concern for adolescents because of the implications of an increasing acceptance by families and peers. This is because waterpipe use is a social activity which can be shared by more than one person, but is less likely to be used at schools because of the equipment required [6]. If waterpipe smoking is occurring it is highly likely to be occurring within families and peer groups at home and in cafes, implying an increasing acceptability and promotion of this practice in young people.

Hence, adolescent smoking behavior is not merely associated with adolescents as individuals, it can also be influenced by the environment they live in including siblings, school, and family. Entry to high school (grade 7) is often accompanied by heightened peer influence [20] and previous studies have found that having a friend who smokes can be a predictor of being smokers during adolescence [23,24]. While peers may influence this process, our study suggests that siblings also have a role. One explanation is that older siblings may be influential role models for younger ones and encourage them to start smoking at a young age [23]. However, the high rates of fathers smoking could be an influential factor as well through the ready availability of cigarettes at home [64]. We note also the difference in schools in ever-smoking, with school with the low socioeconomic status having a significantly higher proportion of ever smokers. While the analyses included some influences such as family smoking patterns, multiple other reasons are likely and required further exploration. One possible reason that we noted in this study was school policy regarding student cigarette smoking. The school that was supported by the USAID had the second lowest rates of “ever cigarette smoking” in the four participating schools. This school has strict anti-smoking policy including surveillance cameras. Given all these factors together, there is an urgent need for family-based interventions [65] and strict age-related policy guidelines for waterpipe cafés and public health campaigns for families. Current policies do not consistently ban cigarette smoking or water pipe smoking in public places in Jordan [66].

This paper contributes to knowledge of adolescent smoking by shedding light on tobacco use among junior high school students with asthma, but more work is still needed among this group who are very vulnerable to tobacco exposure in order to understand this complex bi-directional relationship. Consistent with previous cross-sectional literature, students with asthma in our study were more than twice as likely to report cigarette smoking [28,29,67]. Smoking contributes to asthma [30,31,32,33,34,36,43], and when combined with issues in adolescence in adhering to treatments, exacerbations are likely [68]. Although asthma diagnosis was found to be a predictor of cigarette smoking in our study, it is difficult from a cross sectional study to suggest the direction of the relationship between asthma and cigarette smoking.

This is important to address because of the discrepancy in our findings and previous longitudinal studies, particularly those reporting a decreased smoking incidence among European male adolescents with asthma [43,69]. A possible explanation could be the under-diagnosis and therefore poor management of asthma in Jordan [46,70]. Adolescents in Jordan are often not aware that they have asthma and they report poor quality of life [46,71], which could lead to an increase in smoking incidence [72]. Another possible explanation is the high rates of WP use in our sample and the association with increased cigarette smoking. Furthermore, the cost of such regular cigarette consumption would be expected to be a potential deterrent in adolescents, however, the relatively cheap price of cigarettes in Jordan compared to other countries limits this possibility. Clearly, Jordanian students had easy accessibility to cigarettes from an early age in this context, where, for instance cigarettes can be bought from a variety of shops, even by children, despite local policy prohibiting this practice [8]. Easy accessibility and availability are likely to be factors in the early initiation of smoking, which occurred in primary school for many of the participants, with a substantial increase at high school entry, and consistent with the literature [56,59].

This area has been poorly investigated, despite the high prevalence of adolescents with asthma in Jordan [46], and the high risk of smoking among those with asthma [7]. It is crucial that family physicians consider smoking in every interaction with adolescents, even at a very young age and especially for young people with asthma [11]. Additionally, theoretically sound, school-based peer led educational programs aiming at motivating students to be smoke free should be developed, implemented, and sustained in high schools in Jordan, and particularly for students who have asthma.

One program which offers potential to address this issue is the Adolescent Asthma Action in Jordan [46]. In a recent study in Jordan, this school-based, peer-led asthma and smoking prevention program was implemented for adolescents with asthma. This program was found to be feasible, acceptable, [47] and effective in increasing self-efficacy to resist smoking and asthma-related quality of life among Jordanian adolescent, especially those with asthma and those who reported cigarette smoking [45]. Therefore this program is worth further investigation.

## 5. Limitations

Several limitations of the study need to be acknowledged, primarily that data collection was self-report, raising the question of accuracy of student responses. However, adolescents tend to provide accurate answers in self-administered questionnaires related to cigarette smoking. The study was undertaken in one city and in public schools only which may restrict the generalization of our findings to other parts of Jordan, however, the large similarities in social and cultural attributes across Jordanian regions, and random selection of public schools was a measure used to overcome this limitation and strengthen the study findings. The use of a cross-sectional design for the study limits the implication of the results to association rather than causation, as effects may be bidirectional. Also, cross-sectional findings offer a snapshot of a single moment in time and thus may differ if another time frame had been chosen [73]. Good quality longitudinal studies on large sample are required.

## 6. Conclusions

Cigarette smoking and waterpipe use among early adolescent males in northern Jordan is unacceptably high compared to other countries internationally. More efforts to develop, implement, and sustain policies to control the tobacco epidemic in the Arab world including Jordan is needed. Public health programs are required that address societal and family acceptance of tobacco use in any form and decrease the accessibility, availability and affordability of cigarettes and waterpipe are urgently required. Interventions are needed that target early adolescents and peers, including school-based anti-tobacco smoking interventions to prevent the uptake of tobacco use among this vulnerable age group and create smoke free schools. Particular attention should be paid to students with asthma, who should be targeted by family physicians and in school-based tobacco prevention programs. Longitudinal studies in Jordan are needed to be able to determine if causation relationships exist between asthma and smoking in adolescents, and to be able to design appropriate interventions for Jordanian adolescents who have asthma to help them stay smoke-free and/or decrease the uptake of smoking.
